# Combination of 5-Aminolevulinic Acid and Indocyanine Green in Photodynamic Therapy for Squamous Cell Carcinoma: An In Vivo Study

**DOI:** 10.3390/ijms27177695

**Published:** 2026-08-28

**Authors:** Aisha Mahmood, Jeongwung Seo, Michelle Barreto Requena, Vanderlei Salvador Bagnato

**Affiliations:** 1 Department of Biomedical Engineering, Texas A&M University, College Station, TX 77840, USA; cyh4681@tamu.edu (J.S.); requenamichelle@tamu.edu (M.B.R.); bagnatovs@tamu.edu (V.S.B.); 2 São Carlos Institute of Physics, University of São Paulo, São Carlos, SP 13566-590, Brazil

**Keywords:** photodynamic therapy, squamous cell carcinoma, 5-aminolevulinic acid, indocyanine green, near-infrared light, photosensitizer

## Abstract

Photodynamic therapy (PDT) is a promising minimally invasive treatment for non-melanoma skin cancer (NMSC). Still, its clinical efficacy is limited by light attenuation and drug distribution within tumor tissue, which restricts photosensitizer activation in deeper tumor regions. PDT with 5-aminolevulinic acid (ALA) is widely used as a precursor to induce accumulation of protoporphyrin IX (PpIX), followed by red light irradiation; it is effective for superficial lesions but often fails to achieve complete tumor control in thicker tumors, contributing to long-term lesion recurrence. To address this limitation, we investigated a two-photosensitizer, two-wavelength-PDT approach that combines ALA with indocyanine green (ICG), a near-infrared (NIR)-responsive photosensitizer that can be activated at greater tissue depths. This also promotes different cellular death targets, since ALA-mediated PDT mainly induces direct tumor cell killing through apoptosis and necrosis, whereas ICG-mediated PDT may contribute to treatment effects through deeper tissue activation and vascular-associated mechanisms. In this study, a preclinical model of cutaneous squamous cell carcinoma (SCC) was used, with animals assigned to control, single-photosensitizer PDT (ALA, ICG), and combined photosensitizer PDT treatment groups. The effect of photosensitizer administration and light irradiation sequence on therapeutic efficacy was also investigated, and tumor progression and survival outcomes were monitored over time. The results indicated that the combined ALA+ICG-PDT approach produced the most sustained suppression of tumor growth and significantly prolonged animal survival compared with single-photosensitizer PDT. Importantly, these findings demonstrated a strong dependence on the sequence in which the photosensitizer and light are applied. These findings indicate that integrating photosensitizers activated at complementary wavelengths may improve treatment coverage across the tumor and partially overcome depth-related limitations associated with PDT, representing a promising strategy to enhance PDT efficacy for NMSC and other solid tumors.

## 1. Introduction

Skin cancer, including non-melanoma skin cancers (NMSCs), represents a significant and growing global health concern. According to the International Agency for Research on Cancer (IARC), more than 1.5 million new cases of skin cancer were reported worldwide in 2022 [1]. NMSCs primarily arise from keratinocytes and include basal cell carcinoma (BCC) and squamous cell carcinoma (SCC). Generally, NMSC lesions are slow-growing and most commonly arise in elderly, fair-skinned people in sun-exposed regions such as the face, head, neck, and upper extremities, reflecting ultraviolet (UV) exposure as a major etiological factor. Although many of these lesions are diagnosed at early, superficial stages, effective treatment can still be limited by factors such as tumor heterogeneity, variable tissue optical properties, and insufficient light penetration to achieve uniform therapeutic activation throughout the full lesion depth. These challenges underscore the need for effective, minimally invasive treatment strategies for depth-controlled therapeutic responses in cutaneous malignancies [2,3,4,5,6].

Conventional management of NMSCs, including surgical excision, Mohs micrographic surgery, radiotherapy, and chemotherapy, while effective, is often associated with certain limitations. Surgical approaches may result in cosmetic disfigurement and functional impairment, particularly in anatomically sensitive regions. Radiotherapy can cause radiation dermatitis, fibrosis, and secondary malignancies with cumulative exposure. Additionally, recurrent or aggressive SCC subtypes may exhibit reduced therapeutic responsiveness, highlighting the need for targeted tissue-sparing approaches [7,8,9,10,11].

Photodynamic therapy has gained widespread acceptance for the treatment of premalignant skin lesions such as actinic keratosis (AK), as well as NMSC including BCC and SCC in situ. It is a minimally invasive treatment due to the photosensitizer’s tumor selectivity characteristics and local irradiation, offering favorable cosmetic outcomes and reduced procedural morbidity compared to surgery [12]. Despite already being internationally recommended as an alternative treatment option for these lesions [13,14,15,16], the long-term efficacy, mainly for thicker lesions, remains limited.

### Mechanism and Biological Effects of Using PpIX and ICG in PDT

**Figure 1:**
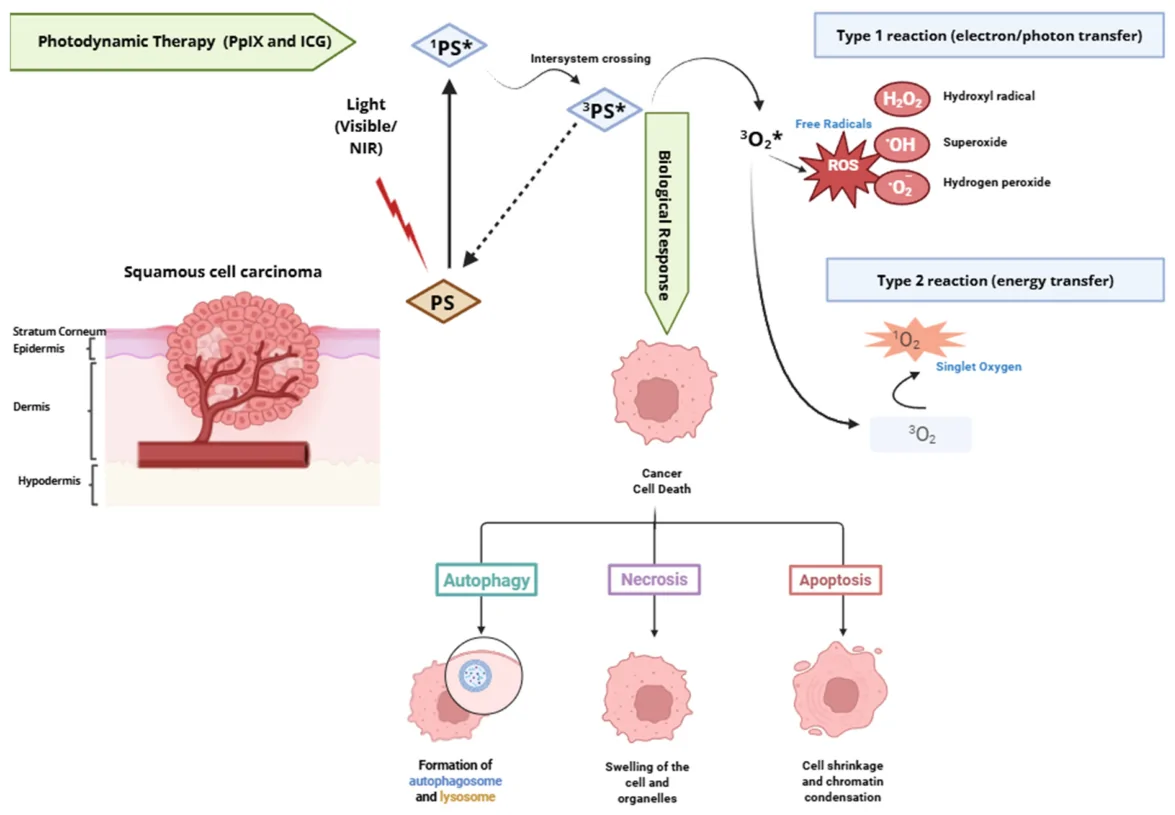
Upon irradiation with visible or NIR light, the photosensitizer (PS) transitions from the ground singlet state to an excited singlet state (^1^PS*), and subsequently to an excited triplet state (^3^PS*) through intersystem crossing. The activated triplet state subsequently undergoes two major photochemical pathways. In the Type I reaction, electron or proton transfer interactions with surrounding biological substrates result in ROS, including hydrogen peroxide (H_2_O_2_), superoxide anion (^•^O_2_^−^), and hydroxyl radicals (^•^OH). In the Type II reaction, transfer of energy from ^3^PS* to molecular oxygen (^3^O_2_) produces singlet oxygen (^1^O_2_), a highly cytotoxic species. The generated ROS, including singlet oxygen, induce oxidative stress-mediated tumor cell death and trigger multiple biological responses, including apoptosis, necrosis, and autophagy. In cutaneous squamous cell carcinoma, ALA-derived PpIX primarily mediates intracellular oxidative damage within tumor cells. In contrast, ICG, activated by NIR light, enhances deeper tissue and vascular targeting, contributing to improved overall PDT efficacy. * Asterisks denote excited states of the photosensitizer or oxygen. Created in BioRender. Mahmood, A. (2026) https://BioRender.com/6mp7y8t, accessed on 29 June 2026 [17,18,19,20].

PDT relies on light activation of a photosensitizer in the presence of oxygen to generate reactive oxygen species (ROS) (Figure 1). Upon irradiation, the photosensitizer absorbs photon energy. It transitions from the ground singlet state to an excited singlet state, followed by intersystem crossing to a longer-lived excited triplet state, which represents the therapeutically active form. The activated triplet-state photosensitizer subsequently interacts with surrounding biomolecules or molecular oxygen through two major photochemical pathways. In the Type I pathway, electron- or proton-transfer reactions generate ROS, including superoxide anions, hydroxyl radicals, and hydrogen peroxide. In the Type II pathway, energy transfer to molecular oxygen (^3^O_2_) produces singlet oxygen (^1^O_2_), a highly reactive cytotoxic species responsible for oxidative damage to proteins, lipids, nucleic acids, and cellular membranes. These photochemical reactions ultimately induce tumor cell death through apoptosis, necrosis, vascular shutdown, or autophagy depending on the intracellular localization of the photosensitizer and the extent of oxidative stress [17,18,19,20]. However, PDT success is constrained by restricted light and drug penetration into tissue, which can prevent adequate photosensitizer activation throughout the full tumor depth [21].

PDT for NMSC commonly uses red light and 5-aminolevulinic acid (ALA, also known as 5-amino-4-oxopentanoic acid) or its derivatives as hydrophilic, non-proteinogenic amino acid prodrugs that feed into the heme biosynthesis pathway to induce intracellular accumulation of the natural photosensitizer protoporphyrin IX [22]. Due to preferential accumulation of PpIX in rapidly proliferating and metabolically active cells, ALA-PDT provides relatively selective tumor targeting while preserving surrounding healthy tissue [17,18]. PDT shows the greatest efficacy in superficial, well-differentiated SCC in situ, including early-stage lesions with minimal dermal invasion [23,24]. Deeply invasive, or perineural, and some poorly differentiated SCC subtypes exhibit reduced responsiveness [25,26] due to limited penetration of visible light as well as heterogeneous oxygen and photosensitizer distribution within tumor tissue [27,28].

Indocyanine green is an FDA-approved NIR fluorescent dye widely used in clinical imaging applications, including retinal angiography, hepatic function assessment, lymphatic mapping, and intraoperative fluorescence-guided surgery for tumor localization and lymph node detection [29,30]. Upon NIR irradiation, ICG generates ROS and localized photothermal effects that can induce tumor cell injury and vascular disruption (following aggregation and polymerization with plasma proteins/lipoproteins). Because NIR light penetrates deeper than visible light, ICG-mediated PDT enables deeper tissue penetration, making it particularly suitable for treating thicker skin tumors while still delivering a dose to the superficial layers. In addition to direct cytotoxicity, ICG-mediated phototherapy can damage tumor-associated vasculature and cause secondary tumor hypoxia. These properties make ICG an attractive photosensitizer for overcoming the depth-related limitations of ALA (PpIX)-mediated PDT in NMSC [31,32].

Based on these considerations, we hypothesized that combining ALA-derived PpIX with ICG for PDT would enhance therapeutic efficacy by complementing the limited tissue penetration of visible-light-activated PpIX with NIR-activated ICG. This two-wavelength strategy was designed to provide complementary photodynamic activity across different tumor depths, thereby improving treatment coverage across the entire tumor depth and potentially reducing the likelihood of residual viable tumor cells. To test this hypothesis, we evaluated the therapeutic efficacy of ALA-PDT, ICG-PDT, and combined ALA+ICG-PDT in an intradermal murine squamous cell carcinoma model.

## 2. Results

### 2.1. Spectral Characterization of PpIX and ICG

**Figure 2:**
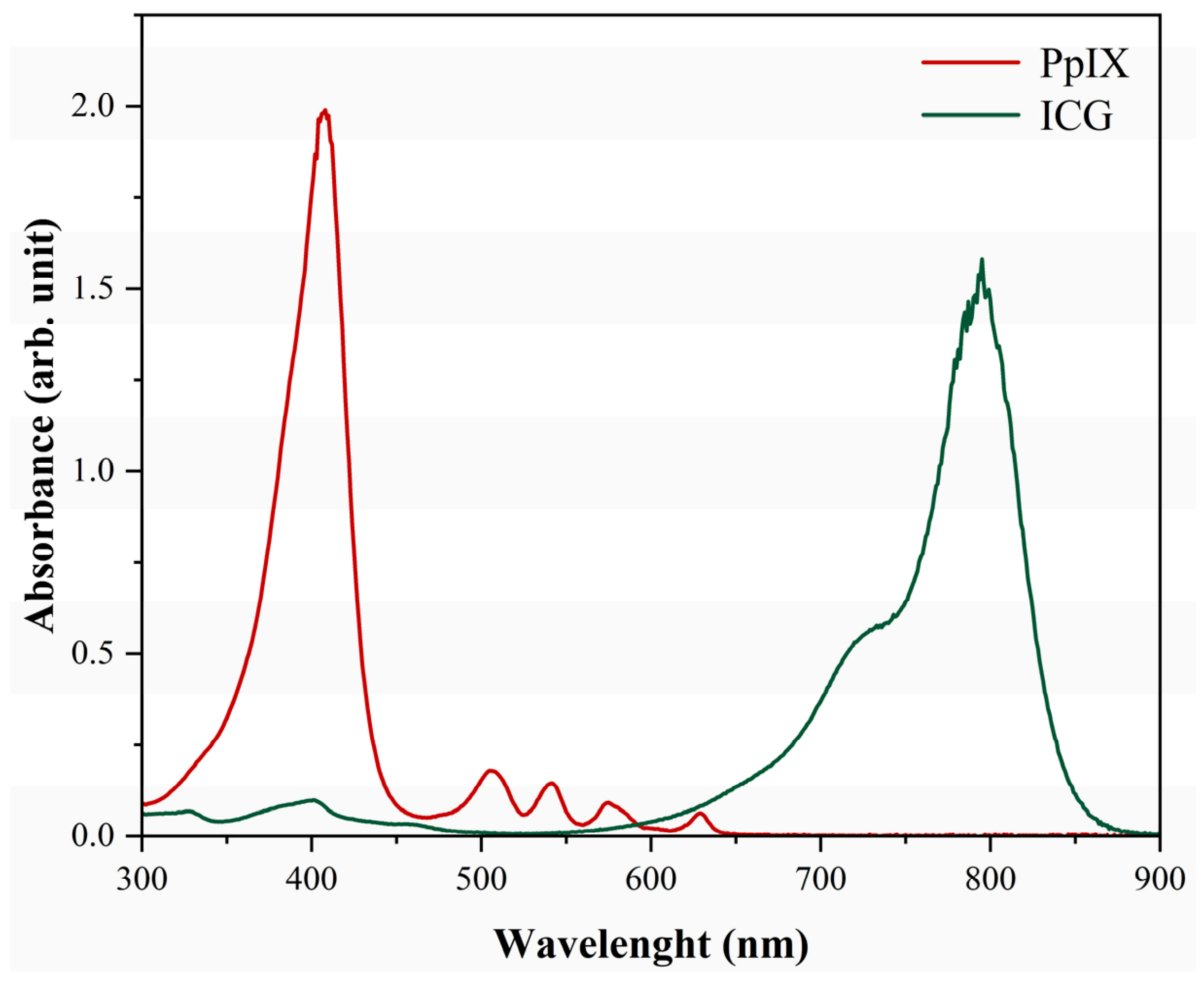
Absorbance spectra of 10 μM PpIX and 10 μM ICG acquired under identical conditions, highlighting the visible absorption of PpIX and the near-infrared absorption of ICG.

The absorption spectra of PpIX and ICG showed distinct and well-separated optical profiles. PpIX exhibited a characteristic porphyrin absorption pattern with a dominant Soret band centered at approximately 405 nm and multiple Q-bands in the visible region, including a peak at approximately 630–635 nm. After wavelengths > 650 nm, the absorbance of PpIX decreased sharply and approached baseline levels in the NIR region. In contrast, ICG showed minimal absorption in the visible range and displayed a strong absorption band in the NIR region, centered at approximately 780–800 nm. This minimal spectral overlap between PpIX and ICG indicates clear spectral separation, thereby enabling wavelength-selective excitation using red light (630 nm) and NIR light (808 nm), respectively (Figure 2).

### 2.2. In Vivo Fluorescence Imaging Confirms Sequential Photosensitization

**Figure 3:**
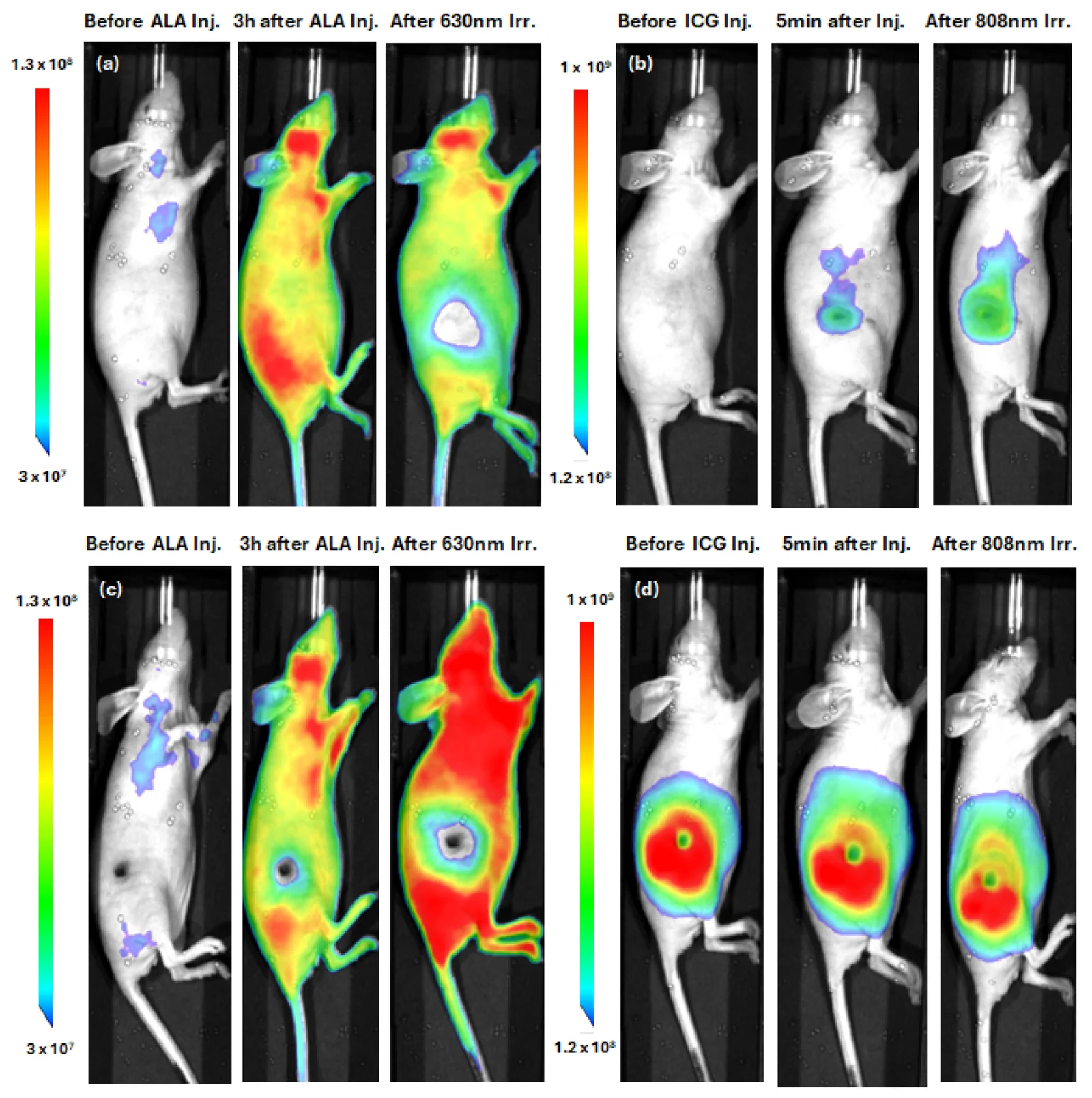
In vivo fluorescence imaging confirming sequential photosensitization. (**a**,**c**) PpIX fluorescence images acquired before ALA administration, 3 h after administration, and immediately after 630 nm irradiation on Days 1 and 2. (**b**,**d**) ICG fluorescence images acquired before ICG injection, 5 min after intratumoral administration, and immediately after 808 nm irradiation on Days 1 and 2. From the images: Inj. = injection and Irr. = Irradiation.

Fluorescence imaging confirmed successful sequential photosensitization following administration of both ALA and ICG in the SCC-bearing mouse model (Figure 3). Before photosensitizer administration, minimal background fluorescence was detected in both PpIX and ICG channels, serving as baseline reference images. Following incubation with each photosensitizer, distinct fluorescence signals became visible in the treated regions, confirming successful accumulation and activation of the photosensitizers before PDT. For the ALA-PpIX channel (Figure 3a,c), fluorescence images were acquired before ALA injection, 3 h after ALA administration, and immediately after 630-nm irradiation on both treatment days. On Day 1 (Figure 3a), strong PpIX fluorescence was observed after the 3-h incubation period, with signal distributed broadly throughout the animal due to systemic administration of ALA. Following 630-nm light irradiation, a noticeable reduction in fluorescence intensity was observed within the treated region, confirming utilization of the photosensitizer during treatment.

For the ICG channel (Figure 3b,d), fluorescence images were obtained before ICG injection, 5 min after intratumoral ICG administration, and immediately after 808-nm irradiation. In contrast to the diffuse PpIX distribution, ICG fluorescence was highly localized to the tumor region following intratumoral injection. Distinct fluorescence signals remained detectable immediately after irradiation, indicating successful tumor-localized photosensitization and retention of ICG within the treated area. On the second treatment day (Figure 3c,d), the same imaging protocol was repeated. PpIX fluorescence intensity was reduced compared with day 1, likely due to a combination of prior photobleaching during PDT and physiological clearance of PpIX over time. In contrast, ICG fluorescence remained strongly localized within the tumor region on day 2. Because ICG exhibits strong optical absorption properties, residual ICG present within the tumor may also have contributed to partial attenuation of the detected PpIX fluorescence signal through optical absorption and shielding effects.

Overall, the fluorescence imaging findings demonstrated successful sequential photosensitization using both ALA-derived PpIX and ICG in the SCC model. The distinct spatial distributions of the two photosensitizers, systemic PpIX fluorescence and localized intratumoral ICG fluorescence, together with the temporal fluorescence changes observed after irradiation, provide direct evidence that PDT was administered under effective photosensitized conditions.

### 2.3. Effect of PDT on Tumor Thickness

**Figure 4:**
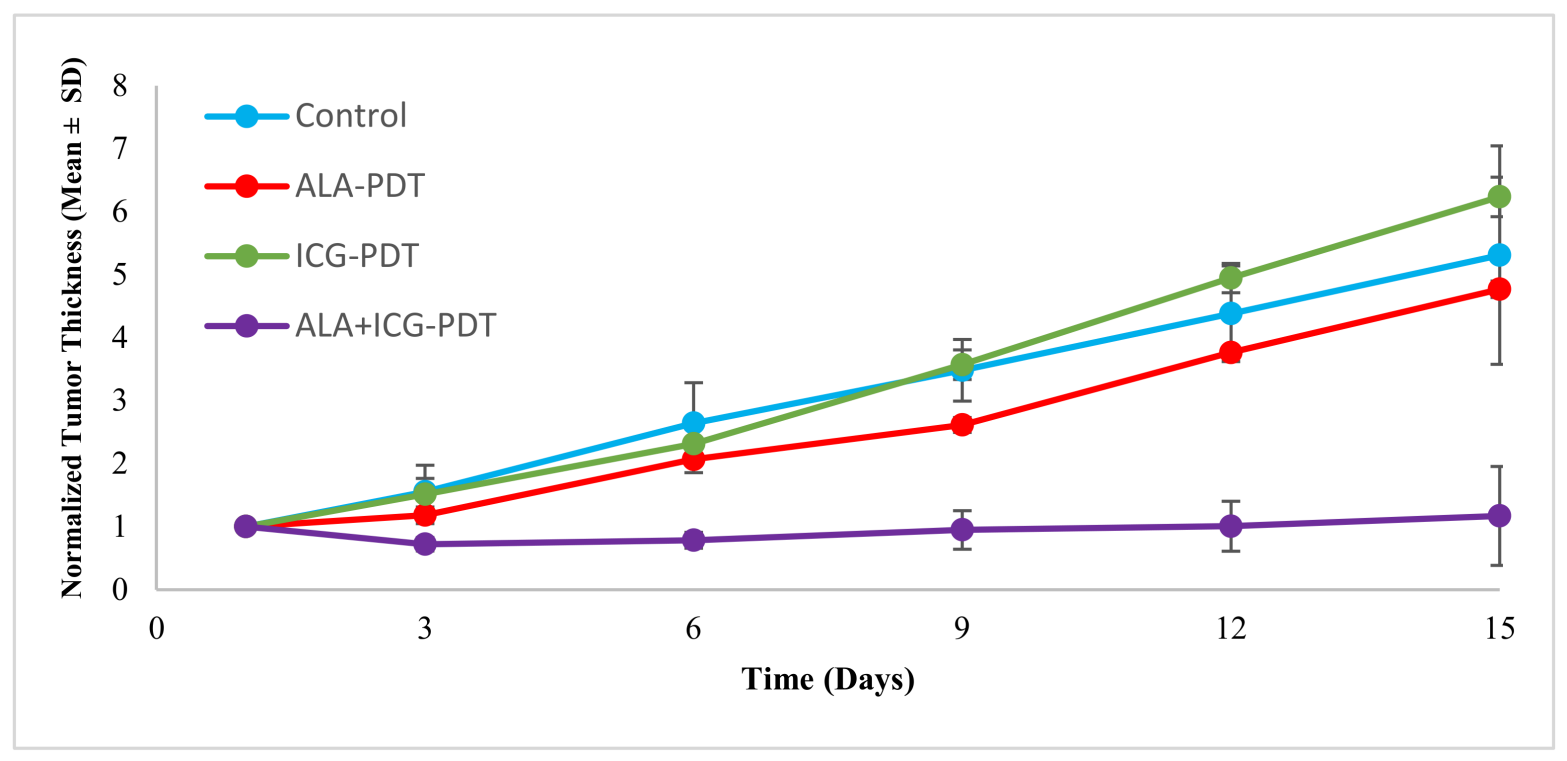
Normalized tumor thickness (mean ± SD) in control, ALA-PDT (630 nm, 120 J/cm^2^), ICG-PDT (808 nm, 120 J/cm^2^), and combined ALA+ICG-PDT (630 nm, 120 J/cm^2^ + 808 nm, 120 J/cm^2^) groups.

Longitudinal ultrasonographic measurements showed progressive increases in tumor thickness in the control group throughout the study period (Figure 4). Treatment with ALA-PDT moderately slowed tumor growth relative to controls. In contrast, ICG-PDT alone exhibited limited therapeutic benefit and showed tumor growth comparable to and exceeding that observed in untreated animals at later time points. In contrast, combined ALA+ICG-PDT produced sustained tumor suppression, with normalized tumor thickness remaining near baseline throughout the observation period. By day 15 (final evaluation time point where data from almost all animals were available), tumors in the combined treatment group exhibited substantially lower thickness values compared with all other experimental groups. These findings indicate that ALA+ICG-PDT provides better tumor progression control relative to a single-photosensitizer treatment approach.

### 2.4. Effect of PDT on Tumor Volume

**Figure 5:**
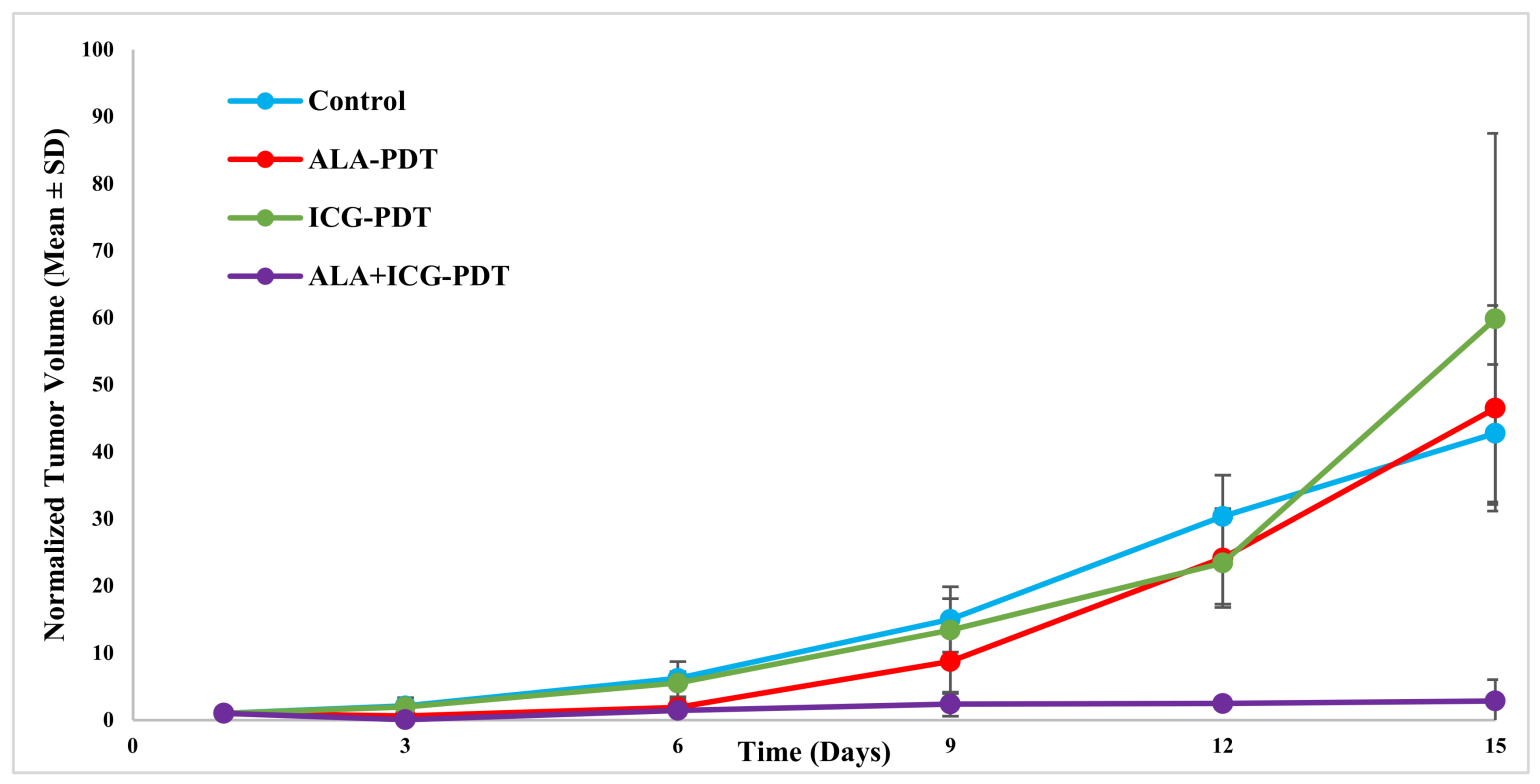
Normalized tumor volume (mean ± SD) in control, ALA-PDT (630 nm, 120 J/cm^2^), ICG-PDT (808 nm, 120 J/cm^2^), and combined ALA+ICG-PDT (630 nm, 120 J/cm^2^ + 808 nm, 120 J/cm^2^) groups.

Similar trends were observed for tumor volume measurements (Figure 5). Control tumors exhibited continuous volumetric expansion throughout the study period. ALA-PDT and ICG-PDT provided limited control of tumor growth, with tumor volumes increasing progressively over time and reaching values comparable to those of the control group by the end of the study. In contrast, animals treated with combined ALA+ICG-PDT demonstrated markedly reduced tumor growth, with tumor volumes remaining close to baseline levels during follow-up. By day 15, the combined treatment group exhibited substantially lower normalized tumor volumes than all other groups, indicating suppression of tumor progression and no tumor regrowth in one animal up to day 90 (Supplementary Figures S1–S3).

### 2.5. Tumor Growth Inhibition Analysis

**Figure 6:**
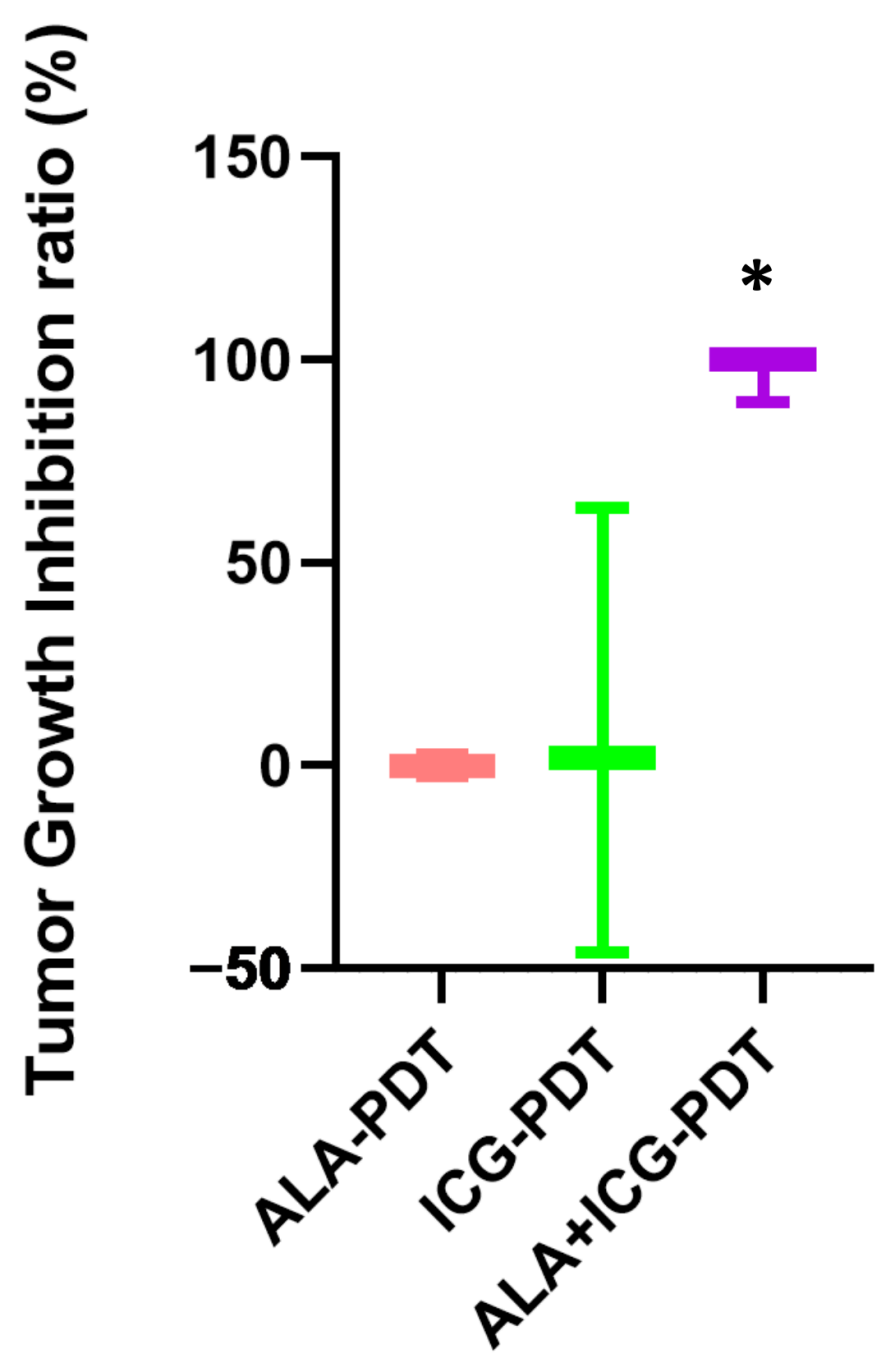
Tumor growth inhibition ratio (%) on day 15 in control, ALA-PDT, ICG-PDT, and combined ALA+ICG-PDT groups. * *p* < 0.05 versus single-photosensitizer PDT group.

Tumor growth inhibition (TGI) analysis also indicated increased therapeutic efficacy of the ALA+ICG-PDT strategy (Figure 6). The combined ALA+ICG-PDT group achieved approximately 84.8% TGI, representing substantial suppression of tumor progression relative to untreated controls. In contrast, ALA-PDT exhibited negative TGI values, indicating tumor growth exceeding that of the control group, whereas ICG-PDT showed only minimal inhibition (approximately 0.3%). Statistical analysis revealed a significant improvement in TGI for the combined treatment group compared with the single-photosensitizer PDT groups (*p* < 0.05). These findings demonstrate that activation of ALA-derived PpIX and ICG when used together provides marked tumor control compared with either photosensitizer alone.

### 2.6. Survival Analysis

**Figure 7:**
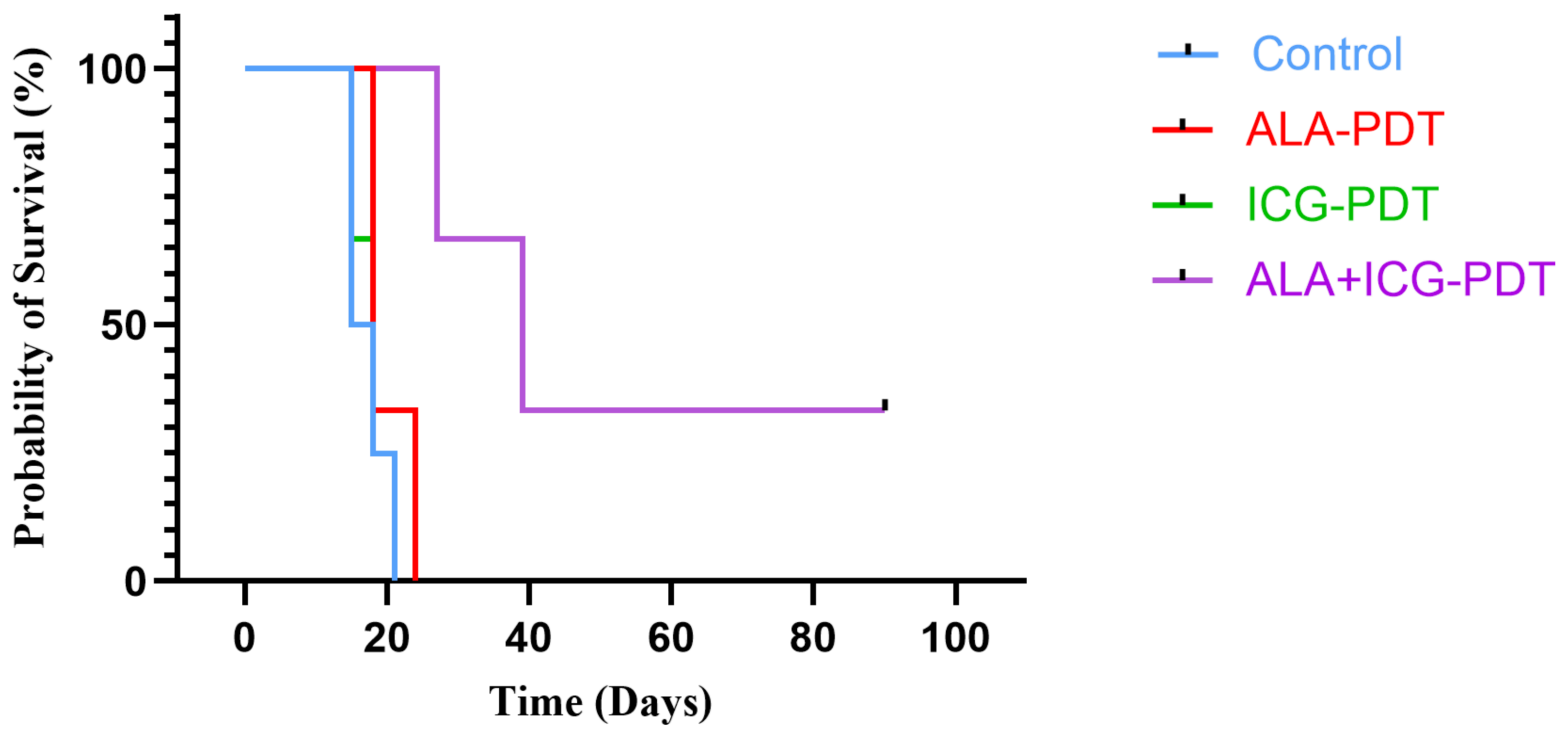
Kaplan–Meier survival curves for control group, ALA-PDT (630 nm, 120 J/cm^2^), ICG-PDT (808 nm, 120 J/cm^2^), and combined ALA+ICG-PDT (630 nm, 120 J/cm^2^ + 808 nm, 120 J/cm^2^).

Results of Kaplan–Meier survival analysis showed poor survival outcomes in control group, with early attainment of humane endpoints (Figure 7). ALA-PDT and ICG-PDT provided limited survival benefits, with survival curves closely overlapping those of the control group. In contrast, the combined ALA+ICG-PDT group exhibited prolonged survival, with a substantial fraction of animals surviving to late time points (up to 90 days). The significant rightward shift in the survival curve confirms that ALA+ICG-PDT not only suppresses SCC tumor growth but also translates into prolonged survival. Collectively, these results demonstrate that while single-photosensitizer PDT provides limited efficacy in this carcinoma model, sequential activation of ALA and ICG produces robust, sustained tumor control and significantly prolongs animal survival.

### 2.7. Functional Validation of Wavelength-Specific Photosensitizer Activation

To confirm that the therapeutic responses observed in the main study result from wavelength-specific activation of each photosensitizer, additional experiments were performed using non-matched illumination conditions. Animals injected with ALA were irradiated using 808 nm NIR light alone and with sequential 808 nm followed by 630 nm illumination. In contrast, ICG-injected tumors were exposed to 630 nm light alone and sequential 630 nm followed by 808 nm irradiation. Tumor growth analysis demonstrated that ALA activated with 808 nm light produced minimal therapeutic effect compared with 630 nm activation. Similarly, ICG exposed to 630 nm light showed negligible tumor suppression. Sequential illumination beginning with the non-optimal wavelength did not substantially improve therapeutic outcomes. These results confirm that PpIX and ICG require wavelength-specific activation, supporting the orthogonal excitation strategy used in the main therapeutic experiments. These supporting experiments (Supplementary Figures S4–S7) confirm that therapeutic efficacy requires wavelength-matched activation of each photosensitizer.

### 2.8. Histopathological Evaluation of SCC Tumors

**Figure 8:**
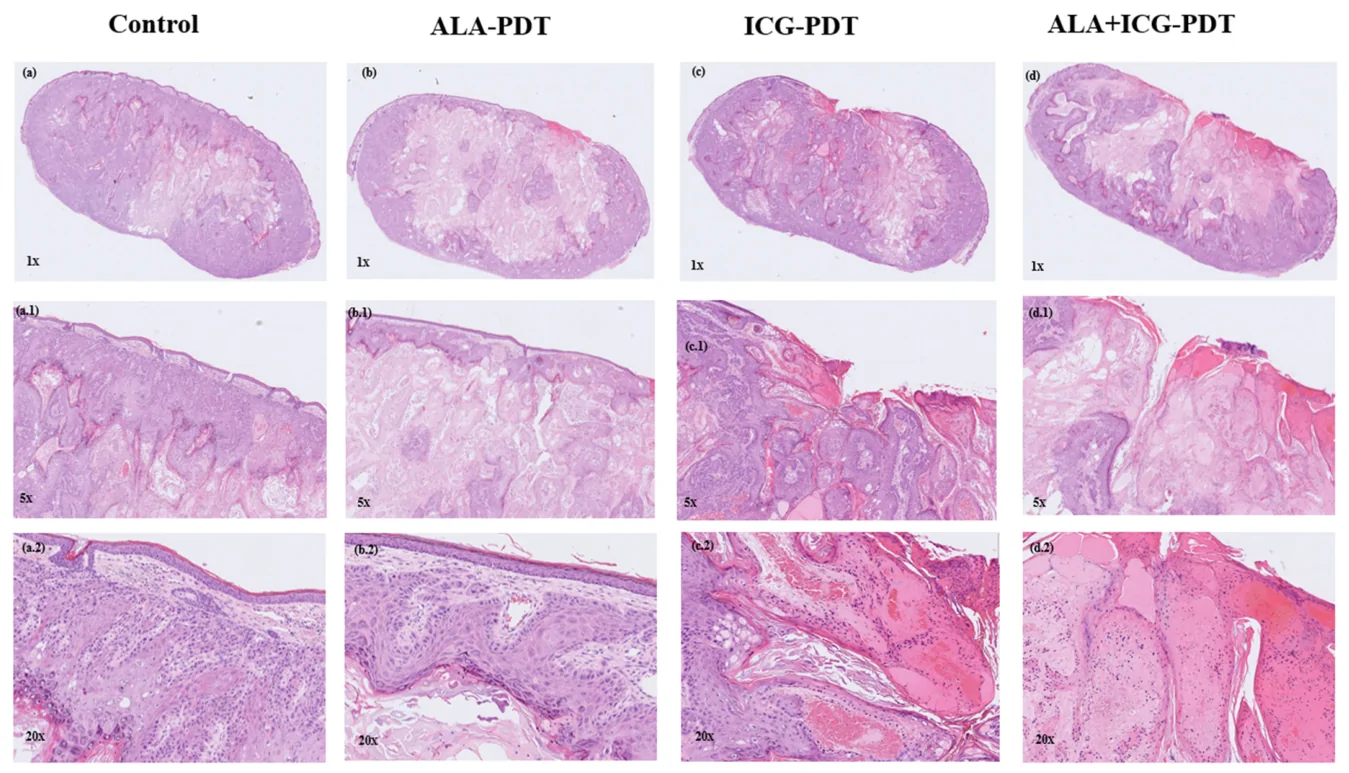
Representative H&E-stained sections of SCC tumors from the experimental groups: (**a**) Control (1×), (**a.1**) Control (5×), (**a.2**) Control (20×); (**b**) ALA-PDT (1×), (**b.1**) ALA-PDT (5×), (**b.2**) ALA-PDT (20×); (**c**) ICG-PDT (1×), (**c.1**) ICG-PDT (5×), (**c.2**) ICG-PDT (20×); (**d**) combined ALA+ICG-PDT (1×), (**d.1**) combined ALA+ICG-PDT (5×), and (**d.2**) combined ALA+ICG-PDT (20×). Hematoxylin stains nuclei dark blue to purple, whereas eosin stains cytoplasmic proteins, extracellular matrix, and connective tissue in varying shades of pink. In necrotic tissue, cells commonly show increased cytoplasmic eosinophilia, loss of normal tissue architecture, and nuclear changes progressing to diminished or absent nuclear staining.

Histopathological analysis of hematoxylin and eosin (H&E)-stained tumor sections revealed distinct morphological differences among the treatment groups (Figure 8). The control group showed typical features of squamous cell carcinoma, including disorganized epithelial architecture, high cellularity, and minimal necrosis. The ALA-PDT group demonstrated partial therapeutic effects, with reduced tumor cellularity and necrosis. In comparison, the ICG-PDT group exhibited more extensive tumor necrosis and fragmentation of the tumor nest. The combination ALA+ICG-PDT group showed the highest histological response, characterized by extensive necrosis and only scattered residual tumor cells. These findings suggest that both ALA-PDT and ICG-PDT reduced tumor burden, while the combination treatment produced the most pronounced histological changes.

## 3. Discussion

### 3.1. Rationale for ALA+ICG-PDT

The therapeutic efficacy of PDT in NMSCs also varies, with early-stage, <2 mm thickness, and <65 years of age patients showing significantly higher response rates compared to advanced tumors [33,34,35]. A major challenge of conventional ALA-PDT in skin cancer is limited penetration of activating light into the tumor lesion, which limits effective photosensitizer activation throughout the entire tumor depth. Therefore, the present study was designed to address this limitation by combining two photosensitizers with distinct optical properties. Spectral characterization confirmed that PpIX and ICG exhibit distinct absorption spectra, enabling wavelength-selective activation with 630 nm and 808 nm illumination, respectively. The strong visible-light absorption of PpIX and the dominant NIR absorption of ICG create a spectrally orthogonal photosensitizer pair that can be independently activated without significant cross-excitation. Such orthogonality is particularly advantageous for multi-wavelength PDT because it allows controlled activation of each photosensitizer within its optimal spectral window.

In addition to spectral separation, the two photosensitizers provide additive photodynamic effects through enhanced depth coverage within tumor tissue. PpIX-mediated PDT primarily targets superficial and intermediate tumor regions due to the limited penetration depth of red light. In contrast, ICG-mediated PDT benefits from deeper tissue penetration of NIR light, which experiences reduced scattering and absorption in biological tissues [31,36,37], consistent with findings that the scattering coefficient decreases with an increase in wavelength [38]. Consequently, combining ALA and ICG expands the effective photodynamic treatment volume by enabling ROS generation over a broader range of tumor depths. This strategy formed the central rationale for the ALA+ICG-PDT approach investigated in this study.

### 3.2. Therapeutic Efficacy in SCC

The therapeutic findings obtained in this study demonstrated a clear advantage of using ALA+ICG-PDT over single-photosensitizer treatments by reducing progressive tumor growth in the combined PDT group compared with the untreated control. In contrast, ALA-PDT produced only moderate tumor growth suppression. Similarly, ICG-PDT alone showed limited therapeutic effect under the selected dose and irradiation conditions. The combined ALA+ICG-PDT produced sustained inhibition of tumor growth throughout the observation period, resulting in lower tumor thickness and tumor volume compared with all other treatment and control groups. These observations were further supported by TGI analysis. At the final evaluation time point, combined ALA+ICG-PDT achieved approximately 84.8% TGI, whereas single-photosensitizer PDT groups showed minimal inhibition. The marked difference in TGI indicates that combining these two photosensitizers, activated at distinct wavelengths, provides a greater therapeutic effect than either modality alone.

Importantly, tumor growth suppression translated into improved long-term outcomes. Kaplan–Meier survival analysis showed that animals receiving combined PDT survived considerably longer than those in the control, ALA-PDT, or ICG-PDT groups. While single-photosensitizer treatments provided little survival benefit, a substantial fraction of animals treated with ALA+ICG-PDT survived to the end of the study period. Histopathological findings further supported these observations, revealing marked tumor necrosis and disruption of tumor architecture in the combination treatment group.

### 3.3. Biological Mechanisms Underlying Enhanced Tumor Control

The superior therapeutic efficacy observed with combined ALA+ICG-PDT may be attributable to complementary biological mechanisms involving ROS-mediated tumor cell killing, vascular damage, and activation of anti-tumor immune responses. ALA-derived PpIX accumulates preferentially within malignant cells and, upon activation with light, generates ROS, including singlet oxygen, that induce oxidative damage to mitochondria, cellular membranes, proteins, and nucleic acids. These photochemical reactions initiate apoptotic and necrotic pathways that contribute directly to tumor destruction. In addition to direct tumor cell killing, PDT exerts anti-tumor activity through vascular destruction and stimulation of anti-tumor immune responses [18,39]. Furthermore, the intradermal tumor environment used in this study is highly vascularized and oxygen-rich, conditions that favor efficient ROS production and may further enhance PDT efficacy [40].

Direct tumor cell killing occurs either by necrosis or apoptosis, where necrotic cell disruption leads to inflammatory reactions. Apoptotic destruction occurs through activated endonucleases, causing DNA damage and leading to activation of caspases. There are two apoptotic mechanisms: 1. intrinsic or mitochondria-mediated apoptosis due to release of cytochrome c and apoptosis-inducing factor in mitochondria, and 2. extrinsic or death receptor-mediated apoptosis, which occurs when photosensitizers target the cell membrane with cell surface death receptors; TNF-receptor (tumor necrosis factor). ROS-mediated photodamage to tumor-associated endothelial cells promotes platelet aggregation, vascular occlusion, and persistent hypoxia and nutrient deficiency, thereby contributing to secondary tumor destruction. Furthermore, PDT-induced tissue injury triggers inflammatory signaling and leukocyte infiltration, including neutrophils and macrophages, which amplify tumor damage and may stimulate systemic anti-tumor immunity. These combined mechanisms make PDT an attractive minimally invasive therapeutic strategy for cutaneous malignancies [18,41,42]. PDT can also influence the tumor microenvironment through induction of inflammatory signaling pathways. ROS-mediated cellular injury promotes release of damage-associated molecular patterns and recruitment of innate immune cells, including macrophages and neutrophils, which may amplify antitumor activation [19,43].

In addition, ICG expands the therapeutic capability of PpIX-mediated PDT by allowing photodynamic activation with NIR light, thereby increasing light penetration and potentially improving treatment efficacy in deeper tumor regions. NIR light experiences reduced scattering and absorption in biological tissues, allowing activation of ICG within deeper tumor regions that may be inadequately reached by 630 nm illumination [31,37]. This enhanced penetration may increase the overall photodynamic effect and thereby improve tumor control. Studies have shown that ALA-PDT primarily induces direct tumor cell killing through cellular targeting, whereas ICG-mediated phototherapy has also been associated with vascular disruption through plasma albumin-mediated targeting. ICG binding to blood albumin may influence its distribution and, upon activation, may contribute to endothelial damage and altered tumor perfusion, resulting in vascular occlusion, ischemia, nutrient deficiency, and secondary tumor destruction [44]. Together, these complementary mechanisms may contribute to the better therapeutic outcomes observed with ALA+ICG-PDT. Although vascular injury was not directly measured in the present study, its contribution to the improved therapeutic response cannot be determined.

Despite the improved tissue penetration provided by NIR light for ICG activation, ICG-PDT alone did not achieve the therapeutic efficacy observed with combined ALA+ICG-PDT under the treatment conditions investigated in this study. PDT efficacy is determined not only by light penetration but also by photosensitizer localization, oxygen availability, intracellular ROS generation, and the biological targets affected by the photosensitizer [45,46]. An increase in the ICG-PDT dose could theoretically enhance treatment effects; however, dose–response optimization was not evaluated in the present study. Instead, PDT generally exhibits a therapeutic window in which treatment outcome depends on the balance among photosensitizer concentration, tissue oxygen availability, and delivered light fluence. Beyond optimal treatment conditions, further increases in light exposure may provide limited additional benefit due to oxygen depletion, photosensitizer photobleaching, and reduced ROS generation efficiency, which restrict further enhancement of photodynamic cytotoxicity. Furthermore, increasing irradiation intensity has been associated with photothermal effects rather than further enhancing PDT-mediated ROS generation [45,46,47]. Therefore, the rationale for combining ALA and ICG was not simply to increase the PDT dose, but rather to explore their complementary properties, including PpIX-mediated direct tumor damage and ICG-mediated deeper tissue activation and vascular-associated effects, to achieve better tumor control through different mechanisms.

ICG is rapidly cleared from the systemic circulation through hepatic uptake and biliary excretion, with a half-life of approximately 3–4 min, which is beneficial in imaging applications [48]. Therefore, ICG in cancer studies is being delivered with nanoparticles. Still, in this study, to maximize local photosensitizer availability and therapeutic efficacy, ICG was administered locally via intratumoral injection [47] to avoid rapid clearance. Long-term outcomes of PDT include local tumor control in superficial NMSC cases, with recurrence rates comparable to surgery in early lesions [24,49,50]. PDT has been reported to partially overcome resistance to conventional chemotherapy or escape resistance pathways and help sensitize tumors to molecular targeted agents. However, resistance and metastasis may still emerge in hypoxic tumors, which are the challenges of PDT, unless otherwise assisted with nanotechnologies, direct exogenous oxygen supply, and regulating the tumor microenvironment [33,34,35].

This study intentionally did not perform exhaustive dose–response optimization for ALA or ICG alone. Therefore, under these controlled but not necessarily optimal conditions, the combined regimen provided superior tumor control compared with the single-agent regimens tested. Future studies should directly compare optimized, higher-dose single-agent PDT with the combination approach and include matched-light-exposure controls to determine whether multi-photosensitizer therapy offers benefits beyond dose escalation alone before clinical translation.

### 3.4. Validation of Wavelength-Specific Activation

An important objective of this study was to determine whether the enhanced therapeutic response observed with ALA+ICG-PDT resulted from wavelength-specific activation of each photosensitizer rather than increased light exposure. To address this question, additional experiments were performed using non-matched wavelength/photosensitizer combinations. When ALA-derived PpIX was irradiated using 808 nm light, tumor growth suppression was minimal, consistent with the negligible absorption of PpIX within the NIR region. Similarly, irradiation of ICG with 630 nm light produced little therapeutic effect because ICG exhibits limited absorption within the visible red spectrum. Sequential irradiation, beginning with a non-optimal wavelength, likewise failed to improve treatment outcomes significantly. These findings are consistent with the spectral characterization results and confirm that efficient activation of each photosensitizer requires illumination within its corresponding absorption band.

The control experiments therefore provide important mechanistic support for combining the two-photosensitizer strategy. The improved tumor control observed in the combined ALA+ICG-PDT group cannot be attributed solely to administration of additional light energy. Instead, the therapeutic benefit arises from orthogonal activation of two spectrally distinct photosensitizers, allowing ROS generation at complementary tissue depths. These findings support the concept that multi-wavelength PDT can overcome depth-related limitations associated with conventional single-photosensitizer therapy [49,51]

### 3.5. Study Limitations and Future Perspectives

The study was conducted in immunocompromised nude mice, preventing evaluation of adaptive immune responses induced by PDT. Second, sample sizes were relatively small (as this study was conducted as proof of principle). Third, mechanistic endpoints including ROS production, vascular damage, and tissue oxygenation were not directly quantified. Future studies should investigate optimized irradiation sequences, photosensitizer dosing, matched-light-exposure controls, and therapeutic efficacy in immunocompetent models and larger preclinical cohorts. Additional investigations evaluating immune activation, vascular responses, and oxygen dynamics would further clarify the mechanisms underlying the therapeutic efficacy of ALA+ICG-PDT.

## 4. Materials and Methods

### 4.1. Photosensitizer for Solution Characterization

PpIX and ICG were prepared as stock solutions and diluted to a final concentration of 10 μM in the appropriate solvent. All samples were freshly prepared before spectral measurements to minimize degradation. The absorption spectrum was recorded using a UV-Vis-NIR spectrophotometer (Agilent Technologies, Santa Clara, CA, USA), and measurements were performed under identical conditions.

### 4.2. Animals and Tumor Model

Female athymic Nu/J nude mice (6–8 weeks old, 20–25 g) were obtained from The Jackson Laboratory (Bar Harbor, ME, USA). All animal procedures were approved by the Institutional Animal Care and Use Committee (IACUC) at Texas A&M University and conducted in accordance with institutional and national guidelines for animal care and use. Animals were housed under specific pathogen-free conditions with a 12 h light/dark cycle and provided ad libitum access to food and water. Following a 7-day acclimation period, intradermal tumors were established on the right flank by injection of A431 human epidermoid carcinoma cells (ATCC; 1 × 10^6^ cells in 30–50 µL sterile PBS) using a 30-gauge needle under inhalation anesthesia (4% isoflurane for induction and 2% for maintenance). Correct intradermal implantation was verified immediately after injection by lateral skin displacement and subsequently confirmed by tumor attachment to and mobility with the skin. Tumors were allowed to grow until reaching an ultrasound-measured thickness of 1–1.5 mm, corresponding to early-stage intradermal lesions suitable for PDT intervention.

### 4.3. ALA and ICG for In Vivo Experiments

ALA (EMI Pharma, São Carlos, SP, Brazil) was supplied as a powder and freshly dissolved in water for injection (100 mg/mL). The pH of the solution was adjusted to 5.0–6.5 using 1 N sodium hydroxide. ALA was administered intraperitoneally at a dose of 200 mg/kg body weight [52,53]. Based on prior PpIX pharmacokinetic studies demonstrating peak intratumoral PpIX accumulation approximately three hours after intraperitoneal ALA administration, a three-hour incubation period was selected before light irradiation [54]. During this incubation period, animals were kept in a dark environment to prevent unintended photoactivation. ICG (Ophthalmos Ltd.a., São Paulo, SP, Brazil) was prepared at 2.5 mg/mL immediately before use and administered via intratumoral injection at a dose of 5 mg/kg [55]. This route was selected to maximize local photosensitizer concentration within the tumor, minimize systemic exposure, and avoid rapid clearance [47].

### 4.4. In Vivo Fluorescence Imaging

In vivo fluorescence imaging was performed to confirm adequate photosensitization of tumor tissues before PDT. ALA and ICG were sequentially and separately administered according to the experimental protocol, and their fluorescence signals were monitored to assess spatial distribution and temporal changes associated with PDT. Fluorescence images were acquired using the KINO imaging system (Spectral Instruments Imaging, Bruker, Tucson, AZ, USA). During imaging, mice were anesthetized with isoflurane and placed inside the light-tight imaging chamber, which also allowed continuous delivery of anesthesia.

For ALA, PpIX fluorescence excitation was selected at 430 nm with a power of 2%, and emission was collected at 630 nm. ICG fluorescence imaging was performed using excitation at 745 nm with a power of 5%, and emission was detected at 830 nm. For both photosensitizers, image acquisition parameters were kept constant across all animals and time points (exposure time: 2 s; binning: 2; object height: 4.5 cm) to allow direct visual comparison of fluorescence intensity. Fluorescence imaging was conducted at three predefined time points on each treatment day: before photosensitizer injection (baseline), after the incubation time (3 h after ALA administration or 5 min after ICG administration), and immediately after light irradiation for PDT. The same imaging sequence was repeated on two consecutive treatment days. Fluorescence images were visualized and processed using the manufacturer-provided AURA version 4.5.1 (Spectral Instruments Imaging, Bruker, Tucson, AZ, USA), and fluorescence intensity was displayed as heat maps overlaid on grayscale real images. Representative images corresponding to the treatment groups were displayed using identical intensity scales (Figure 3).

### 4.5. Experimental Groups and Design

**Figure 9:**
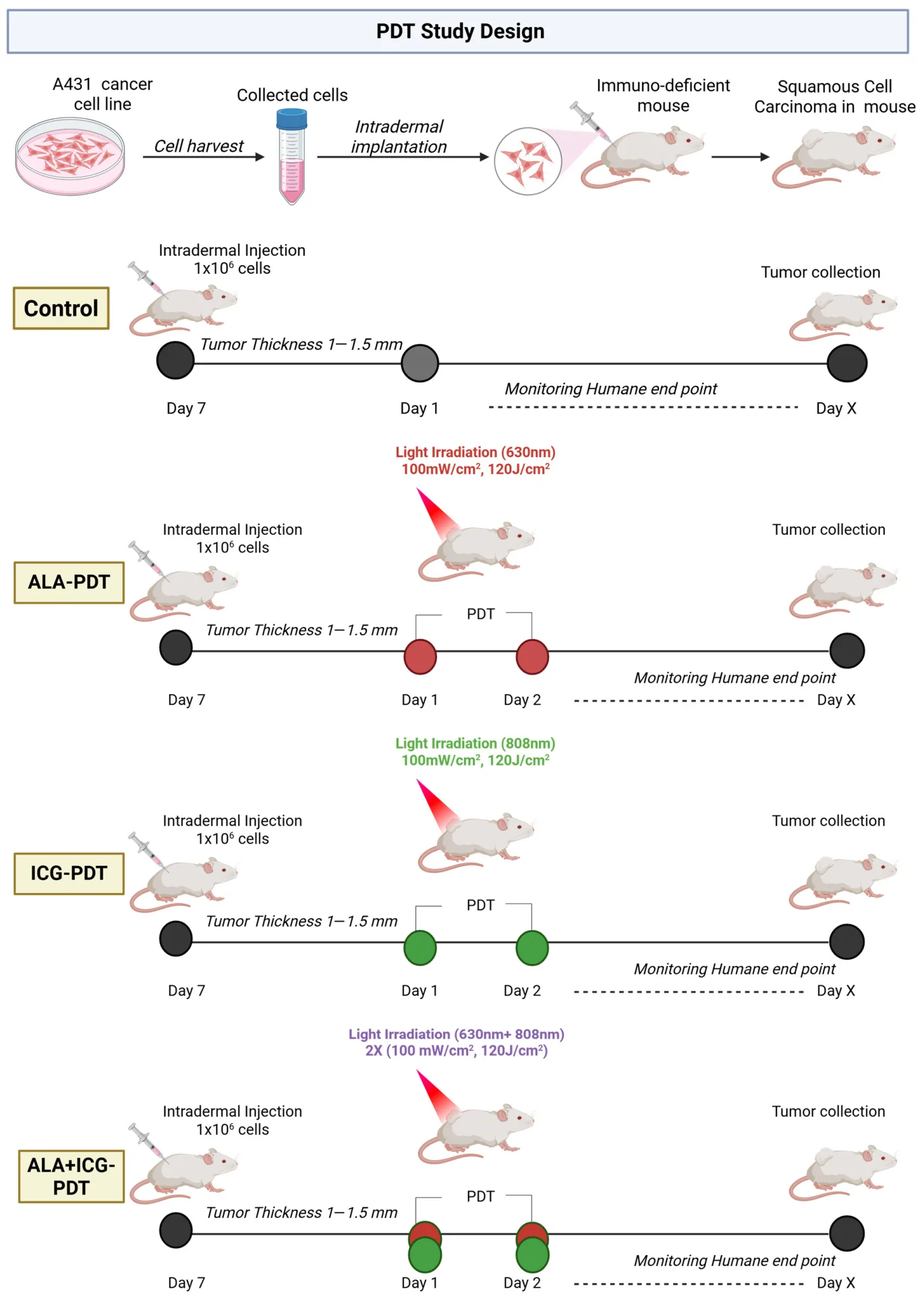
Tumor-bearing mice received ALA-PDT (630 nm), ICG-PDT (808 nm), or combined ALA+ICG-PDT on Days 1 and 2. Black circles represent tumor establishment and tumor collection points; grey circle indicates control (no PDT), red, green, and overlapping circles indicate ALA-PDT, ICG-PDT, and ALA+ICG-PDT, respectively. Created in BioRender. Mahmood, A. (2026) https://BioRender.com/y077vbq, accessed on 8 August 2026.

As illustrated in Figure 9, animals bearing carcinoma tumors were randomly assigned to one of four treatment groups after tumors reached a thickness of 1–1.5 mm as determined by ultrasound imaging (i) control, receiving no photosensitizer administration and no light irradiation; (ii) ALA-PDT, consisting of ALA administration followed by 630 nm light irradiation; (iii) ICG-PDT, consisting of ICG administration followed by 808 nm light irradiation; and (iv) combined ALA+ICG-PDT. All procedures were conducted under general anesthesia, with 4% isoflurane used for induction and 2% for maintenance.

To evaluate the efficacy of ALA+ICG-PDT in SCC, early intradermal tumors with ultrasound-measured thicknesses of 1–1.5 mm were established in female athymic nude (immunocompromised) mice obtained from The Jackson Laboratory (Bar Harbor, ME, USA). The animals were randomized into four groups (*n* = 3/group except ALA-PDT *n* = 2): control, ALA-PDT, ICG-PDT, and combined ALA + ICG-PDT. The ALA was administered intraperitoneally at 200 mg/kg, while ICG was delivered intratumorally at 5 mg/kg. Illumination parameters were selected based on absorbance measurements confirming orthogonal activation of the two photosensitizers, with PpIX being excited by red light and ICG by NIR light. Accordingly, ALA-PDT was performed using 630 nm illumination and ICG-PDT using 808 nm illumination, each delivered at an irradiance of 100 mW/cm^2^ to a total fluence of 120 J/cm^2^. For the combined treatment, both wavelengths were applied sequentially at the same irradiance and fluence per wavelength. As illustrated in Figure 9, animals were allowed to acclimatize for 7 days before study initiation. PDT was performed in two treatment sessions spaced 24 h apart (Session 1 on day 1 and Session 2 on day 2), with ALA administered intraperitoneally 3 h before each treatment session and ICG administered intratumorally 5 min before irradiation. Tumor depth and volume were monitored every third day using three-dimensional high-frequency ultrasonography until humane endpoints were reached. The approved study duration was up to 90 days. However, animals were euthanized upon reaching predefined humane endpoints at different time points during the study.

### 4.6. Photodynamic Therapy Protocol

PDT was performed in two treatment sessions spaced 24 h apart (session 1 on day 1 and session 2 on day 2). For ALA-PDT and combined ALA+ICG-PDT groups, ALA was administered three hours before each treatment session. ICG was injected intratumorally 5 min before irradiation in the ICG-PDT and combined treatment groups. For ALA-PDT, tumors were irradiated with red light at 630 nm. For ICG-PDT, tumors were irradiated with NIR light at 808 nm. Each wavelength was delivered at an irradiance of 100 mW/cm^2^ to a total fluence of 120 J/cm^2^. For the combined ALA+ICG-PDT group, both wavelengths were applied sequentially during the same treatment session, maintaining the same irradiance and fluence per wavelength to ensure independent and orthogonal activation of each photosensitizer. To protect surrounding healthy tissue, the tumor region was isolated using a custom-designed light-shielding mask.

### 4.7. Ultrasound Imaging and Tumor Monitoring

Tumor thickness and volume were monitored every third day using high-frequency ultrasonography (Vevo^®^ 3100 system, FUJIFILM VisualSonics Inc., Toronto, ON, Canada) equipped with an MX550D transducer (40 MHz). Three-dimensional ultrasound images were acquired, and tumor volumes were calculated using Vevo LAB software (version 5.8.1, FUJIFILM Visual Sonics Inc., Toronto, ON, Canada). Animals were monitored until humane endpoints were reached, defined by excessive tumor burden or ulceration, in accordance with IACUC guidelines.

### 4.8. Histopathological Analysis

Excised tumors were collected at study endpoints, fixed in 10% neutral-buffered formalin for 24–48 h, and sent to TVMDL (Texas A&M Veterinary Medical Diagnostic Laboratory) for slide preparation and H&E staining. For histological evaluation, H&E-stained digital slides were loaded into QuPath (version 0.6.0, Centre for Cancer Research and Cell Biology, Queen’s University Belfast, Belfast, Northern Ireland, UK). To capture clean micrographs for presentation, designated areas were exported as high-resolution images.

### 4.9. Randomization

Animals were randomly assigned to treatment groups after tumor establishment. Outcome measurements were performed by the same investigators throughout the study. No formal a priori power calculation was performed because this study was designed as an exploratory, proof-of-concept preclinical investigation.

### 4.10. Statistical Analysis

All data analyses and graph generation were performed using Microsoft Excel for Microsoft 365 (version 2606, Build 2031.20152, Microsoft Corporation, Redmond, WA, USA), OriginPro 2025 (OriginLab Corporation, Northampton, MA, USA), and GraphPad Prism (version 10.6.1.892, GraphPad Software, San Diego, CA, USA). Tumor volumes are expressed as mean ± standard deviation. TGI (%) was calculated at predefined evaluation time points using the following equation:

$$\mathrm{TGI}\;(\%)=\left(1-\frac{V_{t}}{V_{c}}\right)\times 100$$

where $V_{t}$ and $V_{c}$ represent the mean relative tumor volumes of treated and control groups, respectively. Relative tumor volume was defined as the tumor volume at a given time point normalized to the tumor volume measured on day 1. A TGI of 0% indicates no treatment effect; positive values indicate tumor growth inhibition; values approaching 100% indicate complete growth suppression or regression; and negative values indicate enhanced tumor growth relative to control. Statistical comparisons for TGI between groups were performed using one-way ANOVA followed by Tukey’s post hoc test, with *p* < 0.05 considered statistically significant.

## 5. Conclusions

In conclusion, combining ALA-derived PpIX and ICG for PDT significantly improved tumor growth inhibition and survival in a murine SCC model compared with single-photosensitizer PDT. These findings demonstrate the therapeutic advantages of orthogonal activation using visible and NIR wavelengths and support further preclinical investigation of multi-photosensitizer PDT, which may represent a promising strategy to enhance PDT efficacy for NMSCs. This approach may improve treatment efficacy across tumors of different sizes and disease stages while potentially enhancing local tumor control and reducing recurrence in NMSCs. Further studies are needed to evaluate its effectiveness in larger, advanced, and recurrent tumors and to optimize strategies for overcoming hypoxia-associated resistance, thereby facilitating clinical translation.

## Data Availability

The original contributions presented in this study are included in the article/Supplementary Materials. Further inquiries can be directed to the corresponding author.

## Supplementary Materials

The following supporting information can be downloaded at: https://www.mdpi.com/article/10.3390/ijms27177695/s1.
