# Supplementary material for: Combination of 5-Aminolevulinic Acid and Indocyanine Green in Photodynamic Therapy for Squamous Cell Carcinoma: An In Vivo Study
Source: Int J Mol Sci. 2026 Aug 28;27(17):7695. doi: 10.3390/ijms27177695 (PMC13566671; doi:10.3390/ijms27177695)
Supplement: Supplementary file 1 [file ijms-27-07695-s001.zip › ijms-4412404-supplementary.pdf]

Supplementary Materials

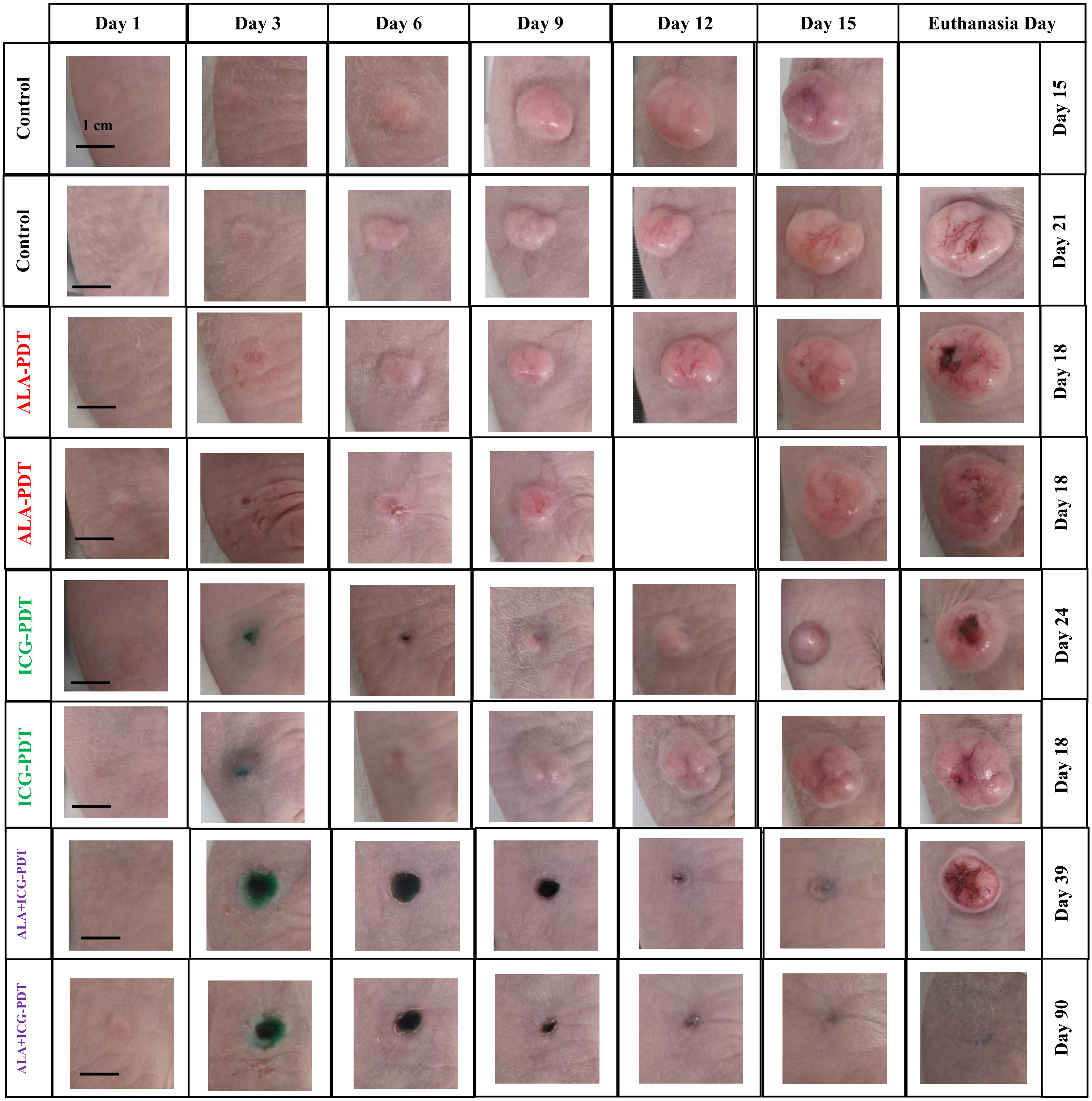

Figure S1: Representative digital photographs of tumors obtained on day 1, day 3, day 6, day 9, day 12, day 15, and at euthanasia. Images illustrate the macroscopic progression of tumor growth and reduction in the Control and treatment groups. Scale bar = 1cm

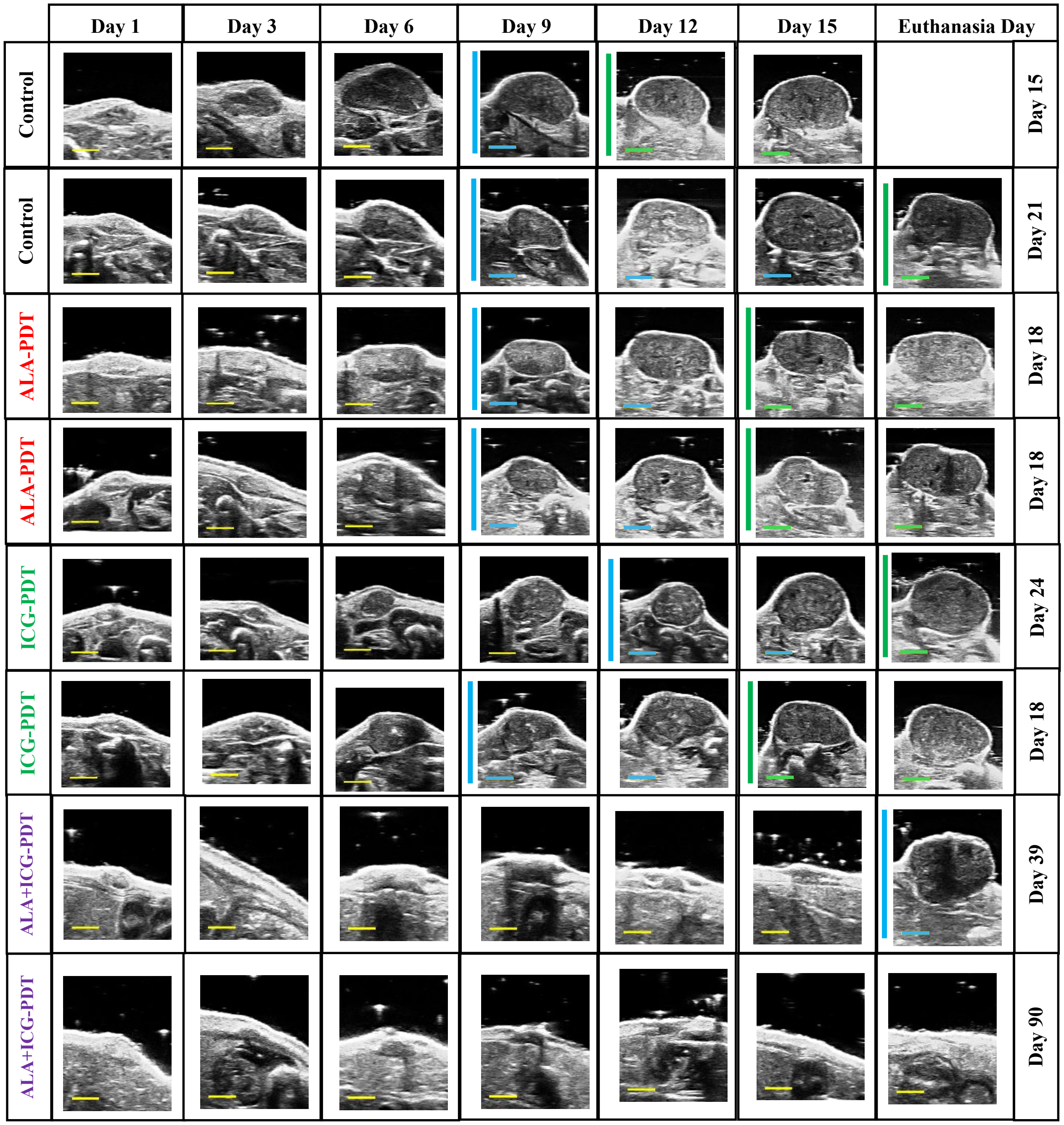

Figure S2. Representative high-frequency ultrasound images acquired longitudinally from day 1, day 3, day 6, day 9, day 12, day 15, and the euthanasia endpoint. Images are shown for the Control group and treatment groups to show tissue changes in tumor thickness and volume throughout study time points. The rightmost column represents images collected at euthanasia for each experimental group. Images were cropped at different field sizes for optimal visualization; scale bars correspond to the crop size used in each image (yellow = 2 mm, cyan = 3 mm, and green = 4 mm).

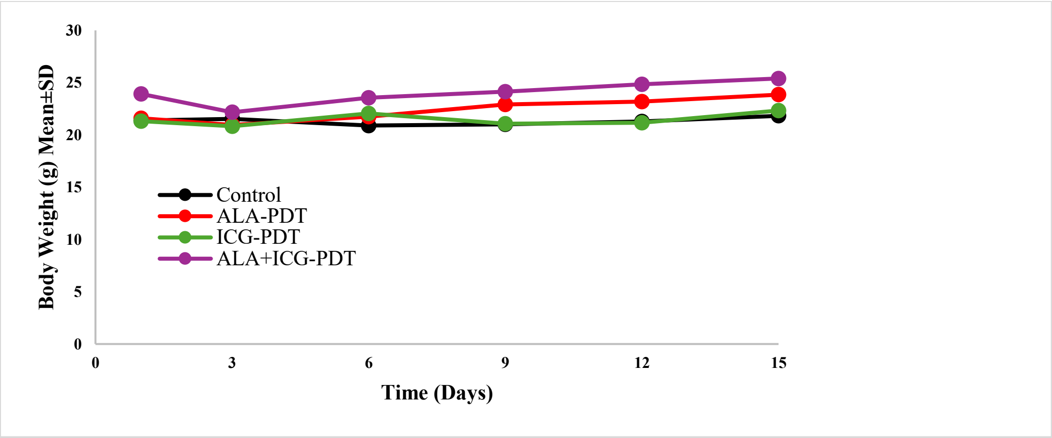

Figure S3: Mean ± SD body weight of mice in the Control, ALA-PDT, ICG-PDT, and ALA+ICG-PDT groups over the 15-day study period. No significant treatment-related body weight loss was observed.

Mismatched PDT.

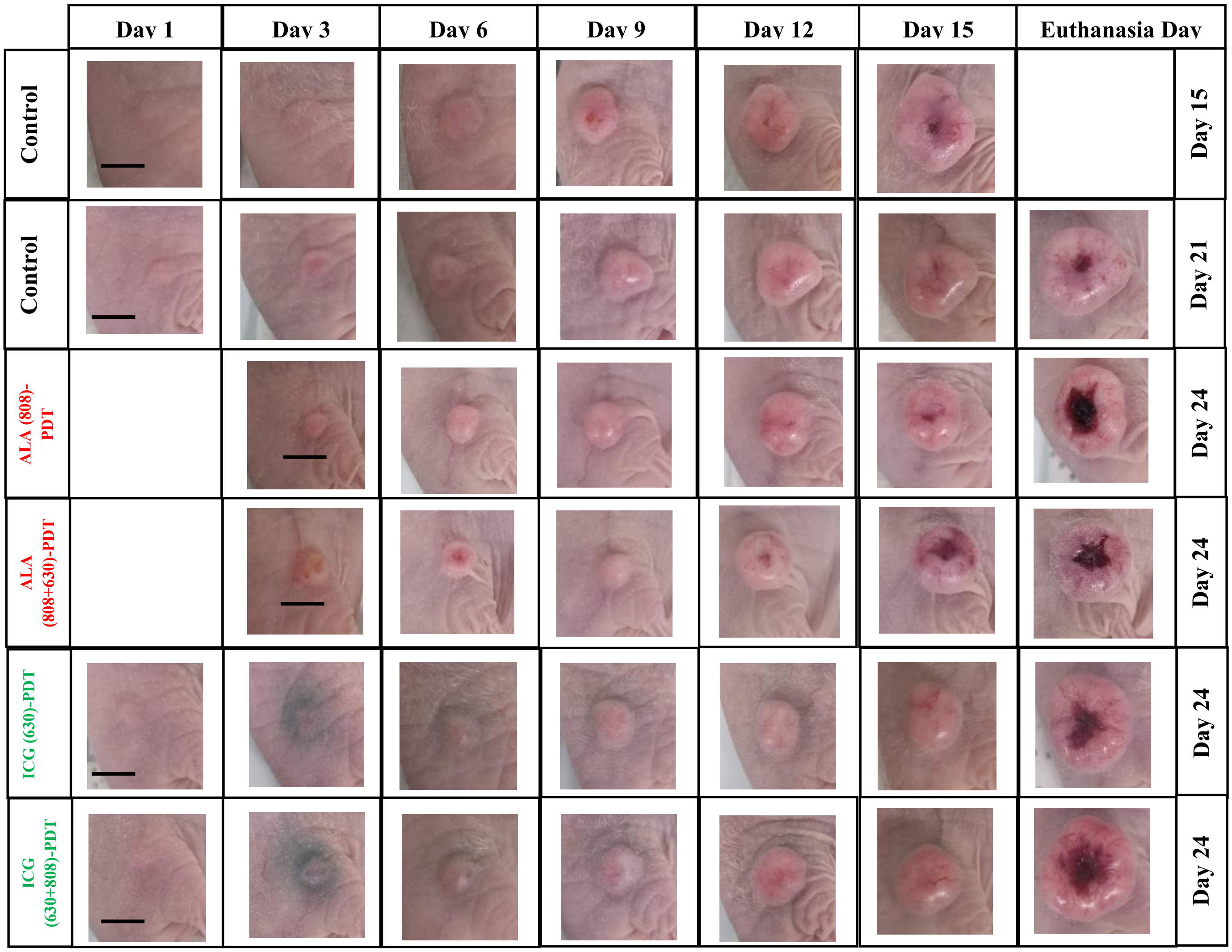

Figure S4. Representative digital photographs of tumors obtained on day 1, day 3, day 6, day 9, day 12, day 15, and at euthanasia. Images illustrate the macroscopic progression of tumor growth and reduction in the Control and treatment groups. Scale bar = 1cm

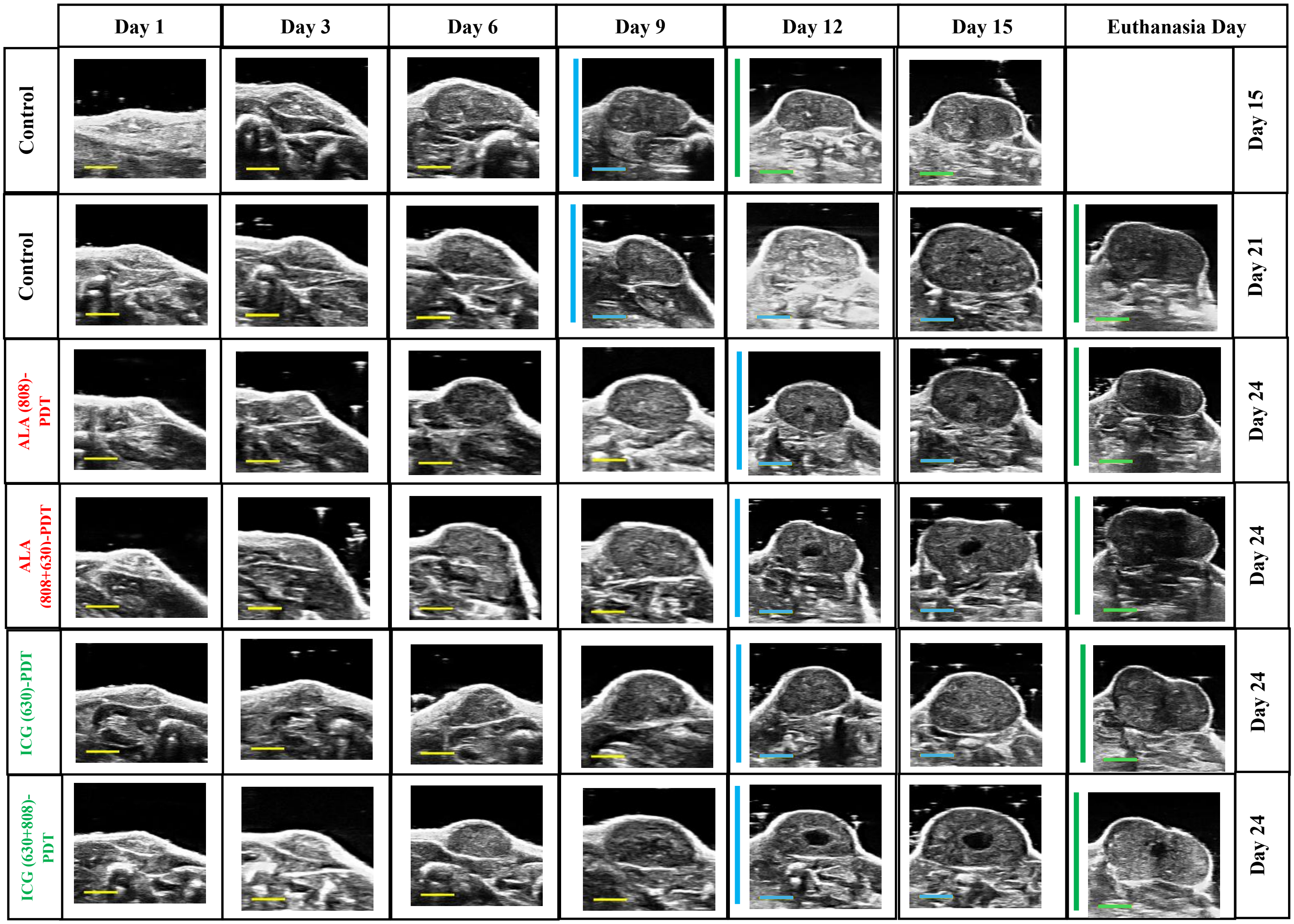

Figure S5. Representative high-frequency ultrasound images acquired longitudinally from day 1, day 3, day 6, day 9, day 12, day 15, and the euthanasia endpoint. Images are shown for the Control group and treatment groups to show tissue changes in tumor thickness and volume throughout study time points. The rightmost column represents images collected at euthanasia for each experimental group. Images were cropped at different field sizes for optimal visualization; scale bars correspond to the crop size used in each image (yellow = 2 mm, cyan = 3 mm, and green = 4 mm).

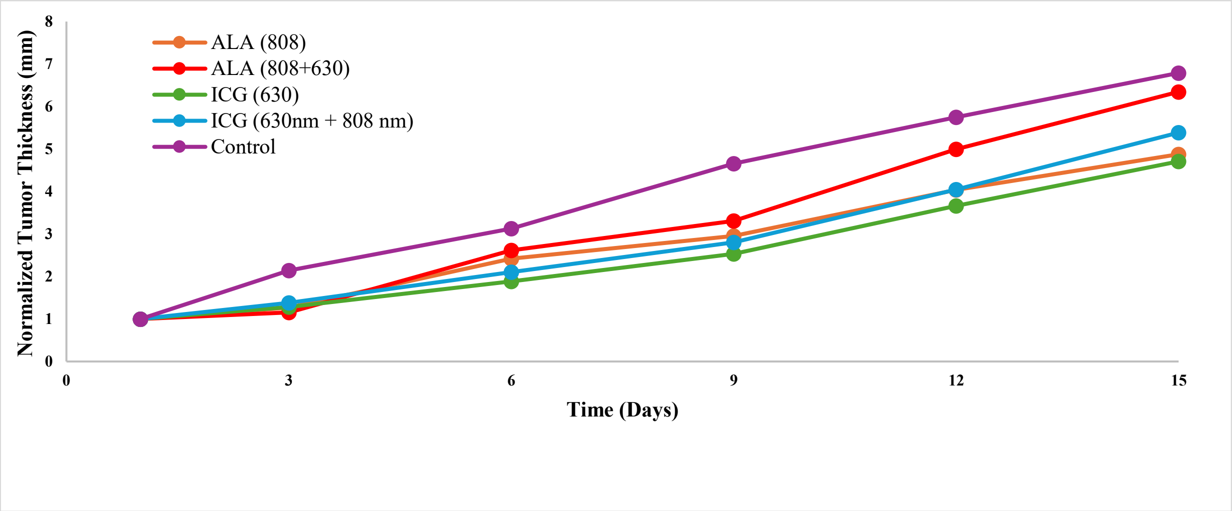

Figure S6: Longitudinal measurements of tumor thickness in carcinoma-bearing mice treated with ALA-PDT at 808 nm, sequential ALA-PDT at 808 nm followed by 630 nm illumination, ICG-PDT at 630 nm, and sequential ICG-PDT at 630 nm followed by 808 nm illumination. Tumor thickness was measured every three days using three-dimensional high-frequency ultrasonography. Data are presented as mean ± standard deviation (SD). These experiments evaluate the therapeutic effect of non-optimal wavelength activation compared with the wavelength-matched PDT conditions used in the main study.

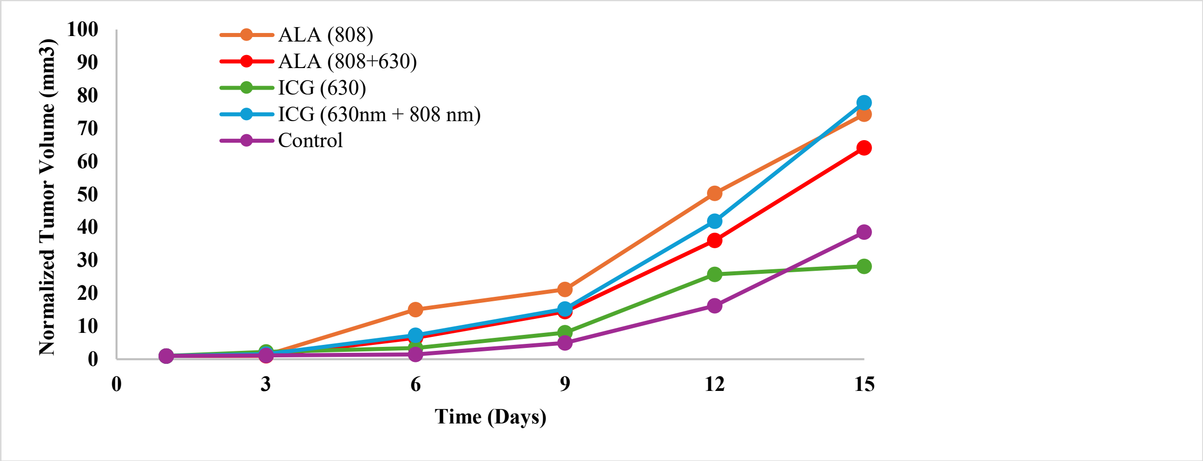

Figure S7: Normalized tumor volume progression in carcinoma-bearing mice treated with ALA-PDT at 808 nm, sequential ALA-PDT at 808 nm followed by 630 nm illumination, ICG-PDT at 630 nm, and sequential ICG-PDT at 630 nm followed by 808 nm illumination. Tumor volumes were calculated from ultrasound measurements and monitored longitudinally throughout the study period. Data are presented as mean ± SD. These results demonstrate limited tumor growth inhibition when photosensitizers are activated with non-optimal wavelengths.
